# Effect of Polyols and Selected Dental Materials on the Ability to Create a Cariogenic Biofilm–On Children Caries-Associated *Streptococcus Mutans* Isolates

**DOI:** 10.3390/ijerph17103720

**Published:** 2020-05-25

**Authors:** Małgorzata Staszczyk, Anna Jurczak, Marcin Magacz, Dorota Kościelniak, Iwona Gregorczyk-Maga, Małgorzata Jamka-Kasprzyk, Magdalena Kępisty, Iwona Kołodziej, Magdalena Kukurba-Setkowicz, Wirginia Krzyściak

**Affiliations:** 1 Department of Pediatric Dentistry, Institute of Dentistry, Jagiellonian University Medical College, 31-155 Krakow, Poland; malgorzata.staszczyk@uj.edu.pl (M.S.); anna.jurczak@uj.edu.pl (A.J.); dorota.koscielniak@uj.edu.pl (D.K.); iwona.gregorczyk-maga@uj.edu.pl (I.G.-M.); malgorzata.jamka-kasprzyk@uj.edu.pl (M.J.-K.); magdalena.kepisty@uj.edu.pl (M.K.); iwona.kolodziej@uj.edu.pl (I.K.); magdalena.kukurba-setkowicz@uj.edu.pl (M.K.-S.); 2 Department of Medical Diagnostics, Faculty of Pharmacy, Jagiellonian University Medical College, 30-688 Krakow, Poland; marcin.magacz@doctoral.uj.edu.pl; 3 Doctoral School of Health and Medical Sciences, Jagiellonian University Medical College, 31-008 Krakow, Poland

**Keywords:** children caries, polyols, cariogenic biofilm, *Streptococcus mutans*, dental materials

## Abstract

Secondary caries is a disease associated with the formation of biofilm on the border of the tooth and dental filling. Its development is strongly influenced by the dietary sweet foods and the type of dental material. The aim of the study was to assess the effect of sweeteners on the ability of clinical *Streptococcus mutans* strains to form biofilm on dental materials. Strains were isolated from plaque samples from 40 pediatric patients from the 3–6 ICADS II group. The ability to form biofilm was tested on composite and glass ionomer dental materials used for milk teeth filling in the presence of sucrose, xylitol, sorbitol, and erythritol. The bacterial film mass after 12, 24, 48, and 72 h and the number of bacterial colonies significantly decreased (*p* < 0.01) compared to the initial value for 5% erythritol and sorbitol on examined materials. A greater inhibitory effect was noted for glass ionomers compared to composites. Sucrose and xylitol supported biofilm formation, while erythritol had the best inhibitory effect. The use of fluoride-releasing glass ionomers exerted an effect synergistic to erythritol, i.e., inhibited plaque formation and the amount of cariogenic *S. mutans*. Selection of proper type of dental material together with replacing sucrose with polyols can significantly decrease risk of secondary caries development. Erithritol in combination with glass ionomer seems to be the most effective in secondary caries prevention.

## 1. Introduction

The development of secondary caries is one of the most common reasons of re-filling dental cavities. This process is associated with the formation of cariogenic biofilm on the boundary between filling edge and a healthy tooth hard tissue and in micro-cracks of dental material. This phenomenon affects the development of the carious process in the surrounding hard tissues, both enamel and dentin, which in turn increases the risk of complications from the pulp and then leads to the need to replace the filling [1,2]. Replacement of the filling is associated with the necessity to widen the removed margins of the tooth tissue, which can then lead to further cycles of intervention and is associated with increased treatment costs [3]. Etiopathogenesis of secondary caries does not differ from primary caries and depends mainly on host-related factors such as diet and oral hygiene [4]. However, according to available in vitro [5] and in vivo [6] tests, the process of plaque formation within a filling is also dependent on the chemical composition of the material (fluoride, chlorhexidine, quaternary ammonium salts) [7] and its physical properties (surface roughness) [8].

Excessive sugar consumption is one of the decisive factors determining the presence of a serious problem that is presence of caries in children all over the world despite technological progress and widely undertaken actions to promote oral health [9,10]. Therefore, research is more and more often conducted on health-promoting aspects of food products that complement classic nutrition regimens and are a good alternative to products containing simple sugars, especially sucrose [11]. The ingredients corresponding to the taste of sucrose are polyols, such as xylitol (100% sweetness of sucrose), sorbitol (50–70% sweetness of sucrose) and erythritol (60–80% sweetness of sucrose) [12,13].

These substances are naturally found in many fruits and are manufactured from natural raw materials, such as lignocellulosic biomass (xylitol), starch (sorbitol), and glucose (erythritol) [14,15]. In vitro, in vivo, and clinical studies have consistently demonstrated the safety of these substances [1,2,3,4], which has been confirmed by recommendations issued by the WHO/FAO (World Health Organization/Food and Agriculture Organization of the United Nations) Joint Committee of Experts on Food Safety in over 60 countries around the world [16].

**Figure A1:**
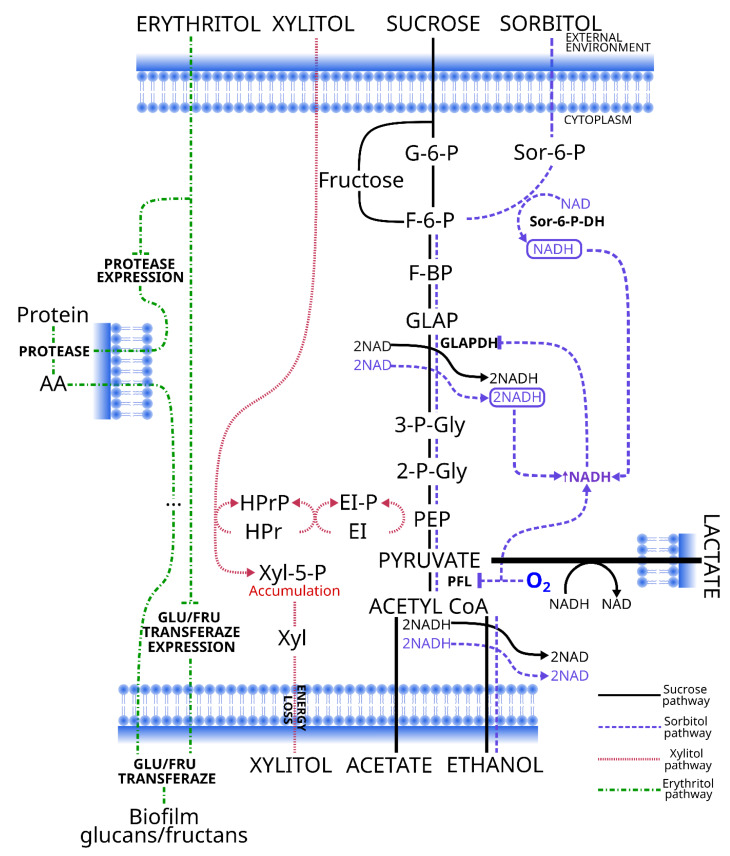
Metabolic pathways of particular sweeteners in the *S. mutans* cells and the inhibitory mechanism of polyols on the metabolism of microorganisms.

It should be noted that the replacement of cariogenic sugars with polyols has a broader significance than just passive inhibition of the metabolism of dental plaque by limiting the access of substrates. There is evidence that xylitol, sorbitol, and erythritol can actively inhibit dental plaque growth by integrating into microbial metabolism and supporting oral health [5,6,7]. Influence on microbial metabolism of each polyol and sucrose is illustrated on Figure A1 in Appendix A section.

Basic research aimed at seeking potential new agents for the use of caries prophylaxis by inhibiting the development and metabolism of cariogenic biofilms is important in the face of the non-decreasing problem of childhood caries [1,17,18,19]. In addition, knowledge about the relationship between the development of secondary caries and diet [20], as well as the reduction of cariogenic sugars’ (e.g., sucrose) consumption, is important to develop new strategies for preventing this disease and plan healthcare in particular groups of patients.

Fluoride prophylaxis in the form of toothpastes, mouth rinses, professional fluoride treatment, and dental cavity filling with fluoride-releasing materials is a widely used strategy to prevent caries. However, due to the large role of dietary factors in its pathogenesis, it is important to replace sucrose-rich foods with foods lacking simple sugars or with natural sucrose substitutes such as xylitol, sorbitol, or erythritol, additionally characterized by cariostatic properties [16,21].

A wide range of knowledge gaps exist according to secondary caries. Despite the fact that it is defined as a lesion associated with restorations or sealants, the impact of the restorative material itself on the secondary caries, based on randomized controlled trials [22,23,24], seems to be limited. The influence of the restorative materials on secondary caries has been evaluated most frequently. The available in vitro studies found no significant difference between various composites on the demineralization of surface and wall lesions. Some studies revealed that fluoride-containing materials (e.g., glass ionomers) reduce secondary caries progression [25,26,27]. A recent systematic review, of in situ studies, could not identify significant differences in secondary caries development in surrounding hard tooth tissues when different materials were used [28]. Clinical studies compared materials for their risk of secondary caries, but only limited data from randomized controlled trials provide evidence for the comparison between composites and amalgam. Glass ionomers show similar effectiveness regarding to secondary caries in comparison with composites but may be connected with higher risk of fracture in extended cavities [29].

Similarly, the hypothesis that the most frequent surface location of secondary caries is the gingival margin of restorations has not been confirmed by available data [30]. While the caries risk or susceptibility of a patient seems to be the most relevant factor for secondary caries development, the risk of restoration failure due to secondary caries is significantly increased in high-risk caries patients compared with low-risk ones in available literature [6,31,32]. Moreover, factors such as the presence and size of restoration gaps or experience of the operator seem to play an important role in secondary caries development but need to be confirmed in in vitro and clinical studies.

What we know for sure is that the factors that cause demineralization, which are related to an individual behavior misbalance on sugar consumption associated with local biofilm accumulation, are exactly the same for primary and secondary caries [29,33].

In addition, there is a gap in the literature regarding the study of the behavior of dental materials in relation to the development of secondary caries in the presence of cariogenic sugars and/or polyols. Therefore, the purpose of our research was to assess the impact of commonly used dental fillings in the presence of sugar and its substitutes on the ability to form *Streptococcus mutans* biofilm.

## 2. Materials and Methods

### 2.1. Ethical Aspects

The study was conducted in accordance with the 2013 Helsinki Declaration. The bioethical commission (consent No. 1072.6120.183.2017) of the Jagiellonian University (Kraków, Poland) approved the study protocol. Patients and their legal guardians with full legal status expressed written informed consent to participate in the study. Patient’s personal data has been anonymized.

### 2.2. Participants of the Study

The study was conducted in 2019–2020 at the Department of Pediatric Dentistry at the University Dental Clinic in Kraków and involved 40 patients aged 4–9 years diagnosed with caries. The study was conducted based on the criteria established by the World Health Organization for epidemiological studies.

The group included generally healthy children with caries defined as cavitation change, i.e., code 3–6 according to the ICDAS II classification (Coordination Committee for the International Caries Detection and Assessment System, 2011) [34].

### 2.3. Dental Plaque Collection

Fasting plaque samples from children were collected at 8–9 a.m. according to the study protocol proposed by Krzyściak et al. (after morning tooth brushing and mouth rinsing with deionized water for 30 s) [35]. Plaque from all tooth surfaces was collected using sterile machine toothbrushes, which were then placed in 1 mL sterile saline at pH = 7.0 at a room temperature. Samples were transported within 4 h to the microbiology laboratory.

### 2.4. Laboratory Analyses and Proceeding with Dental Materials

Two dental materials most often used in patients of developmental age in the Department of Pediatric Dentistry were used in the study. EQUIA^®^ Forte Fil glass ionomer (GC, Tokyo, Japan) was used with a consideration of quickness of work and procedures, as well as high fluoride release. Ceram X^®^ composite (One Universal, Dentsply, DeTrey, Konstanz, Germany) was used while taking into account aesthetics, strength, and medical experience with this material in children.

Materials were prepared and developed on the surface of round slides of a 12 mm diameter according to the manufacturer’s instructions for each of the selected materials. Samples of Ceram X^®^ of thickness not exceeding 2 mm were irradiated with a 1200 mW/cm^2^ polymerization lamp (GC D-Light Duo) for 20 s. Before preparing the glass ionomer samples, the material capsules were mixed for 10 s. After the recommended setting time of 2.5 min, the samples were coated with EQUIA^®^ Forte Coat varnish and light-cured using a diode polymerization lamp for 20 s [36].

Prepared materials were stored under conditions imitating moist oral environment with reduced oxygen availability for 24 h at 37 °C to allow complete polymerization of composite and glass ionomer. Slides were sequentially sterilized in a plasma sterilizer for one hour at 45 °C to fixate the resin surface.

To assess the homogeneity of the material surface, their surface roughness was measured using a needle profilometer (Bruker DektakXT, Breme, Germany) after incubation in the test medium with sugars under sterile conditions.

Materials fixed on slides were then transferred to sterile microtiter plates containing 2 mL of sterile PBS in each well and stored at room temperature for another 10 days to allow unreacted monomers to leach from slides coated with dental materials.

### 2.5. Microbiological Analysis

Plaque samples were prepared for analysis by gentle centrifugation and sonication for 30 s. Serial dilutions of the stock solution in sterile saline were then prepared. The resulting dilutions were inoculated on 10% blood agar plates and selective media used to detect certain groups of microorganisms described by Krzyściak et al. [35].

Inoculated media were incubated in microaerophilic conditions in the presence of 5% CO_2_ at 37 °C for 24–48 h. The quantity and morphology of individual species of microorganisms were assessed.

Species identification was carried out based on the MALDI-TOF system combined with mass spectrometry (MS) (Bruker Daltonik, Germany). Identification of microbial species was possible by comparing obtained protein profiles with molecular mass, charge, and time-of-flight distribution spectra with reference spectra from the database (MALDI Biotyper 3.0 software, Bruker Daltonik, Bremen, Germany). The probability of correct identification was expressed as a point indicator, whose value was set at ≥2000.

### 2.6. In Vitro Biofilm Formation

Microbiological assessment of the ability of biofilm formation by 40 clinically isolated *S. mutans* strains was performed on 24-well sterile flat-bottomed microtiter plates with selected dental materials with 12 mm diameter polystyrene slides placed on the bottom of the wells.

Prepared samples of composite materials and glass ionomer were placed in 2.0 mL BHI culture medium with 5% sucrose, 5% xylitol, 5% sorbitol, or 5% erythritol (Merck, Poland) containing the suspensions of bacterial strains. They were then incubated for 72 h at 37 °C under microaerophilic conditions in a 10% CO_2_ atmosphere. Culture medium was inoculated with the test suspension of pure bacterial cultures in the logarithmic phase of bacterial growth at a concentration of 1 × 10^7^ cells/mL.

### 2.7. In Vitro Biofilm Model

**Figure 1:**
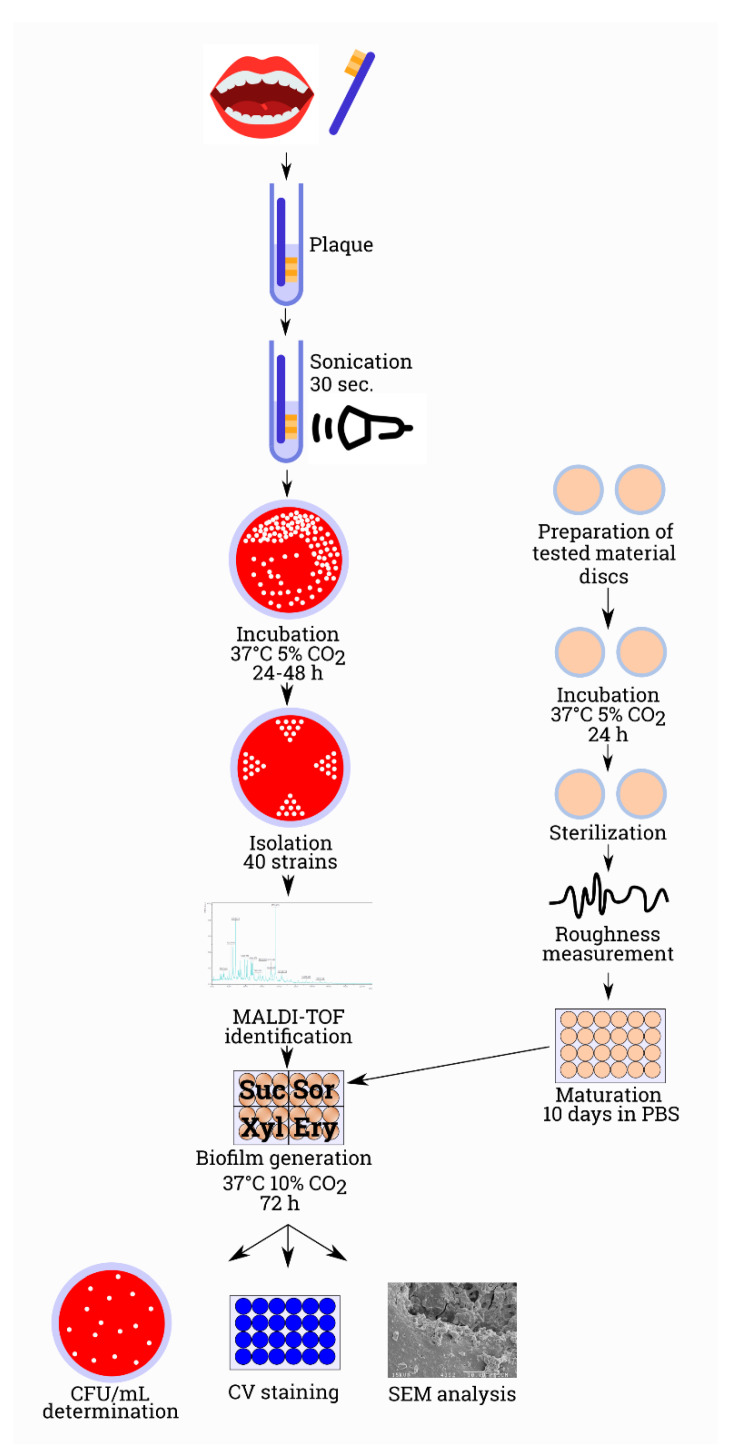
The course of the experiment from the moment of sample collection to the individual determinations.

The ability to form biofilm by the tested isolates (biomass, number of colonies in the biofilm, biofilm formation analysis in a scanning electron microscope) on selected dental materials and under the influence of the sweeteners was assessed in accordance with the method described in our previous work [35]. Figure 1 illustrates the process of the experimental procedure.

### 2.8. Statistical Analysis

Data were analyzed with a significance level set at α = 0.05. Material surface roughness, biomass, and biofilm production were analyzed using ANOVA. Descriptive statistics were calculated, i.e., mean value and standard deviation for variables with normal distribution and median and quarter deviation ((Q3-Q1)/2) for variables with non-normal distribution. The compliance with normal distribution was assessed by Shapiro–Wilk test, the homogeneity of variations in the groups–by Levene’s test, the assumption of the sphericity of variance for the repeated measurements–by Mauchly’s test. ANOVA test and Tukey’s post-hoc analysis were used to assess the differences between the groups (dental material, type of sweetener). Friedman’s test with Bonferroni’s post-hoc test was used to analyze the differences between repeated measurements (12, 24, 48, 72 h), and strength of the effect was calculated by Kendall’s W coefficient, which assumes a value from 0 (indicating no relationship) to 1 (indicating a perfect relationship). Kendall’s W uses the Cohen’s interpretation guidelines of 0.1–0.3 (small effect), 0.3–0.5 (moderate effect) and ≥ 0.5 (large effect). Statistical analyses were carried out in the R 3.5.2 environment [37], and charts were prepared using the ggplot2 package.

## 3. Results

### 3.1. Microbiological Identification

Isolation and identification of *S. mutans* strains from dental plaque were performed using the MALDI-TOF MS technique described in the Section 2.5. Microbiological analysis.

### 3.2. Surface of Tested Materials

The results regarding the surface of the examined dental materials are presented in Table 1. The roughness of the resin-based composites (RBC) was significantly lower compared to the glass ionomer (*p* ≤ 0.01). The lowest surface roughness was presented by the control surface, i.e., a polystyrene disk not covered with any material.

### 3.3. Biofilm Biomass Assessment

**Figure 2:**
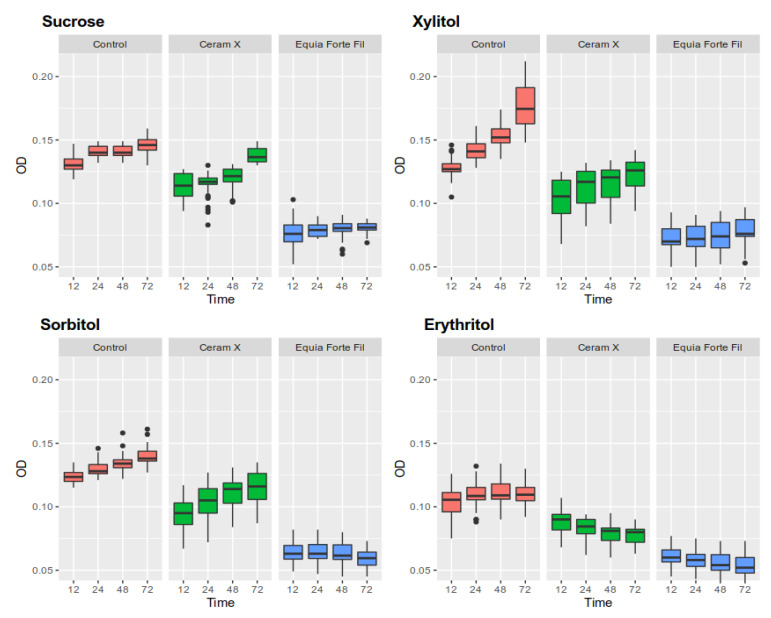
Total mass of biofilm on particular dental materials and the control medium produced under the influence of 5% sweeteners at different time-points.

**Figure 3:**
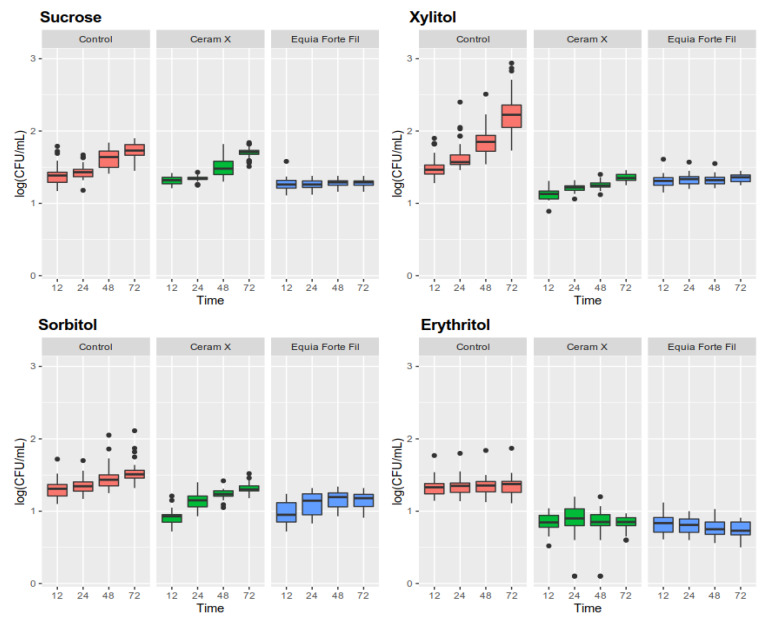
The amount of live microorganisms in biofilms on particular dental materials and the control medium produced under the influence of 5% sweeteners at different time-points.

The results of monospecies biofilm formation (biomass, number of biofilm colonies, structure, and morphology) of *S. mutans* were analyzed on 40 samples of each dental material, i.e., EQUIA^®^ Forte Fil glass ionomer and Ceram X^®^ composite. There were statistically significant differences (*p* < 0.0001, ANOVA) both between the biofilm biomass and the number of biofilm-forming microorganisms relative to control (both on composites and glass ionomers). A statistically significant slowdown in biofilm formation (both biomass and biofilm colony count) was observed for both dental materials compared to controls (discs not covered with test materials) at all time-points (12, 24, 48, 72 h). The same dependence was observed for the samples containing 5% polyols in relation to 5% sucrose. The results are presented in Table 2, Table 3 and Table 4 and Figure 2 and Figure 3.

### 3.4. Measurement of Total Biomass Expressed as OD (Optical Density)

Erythritol was characterized by the strongest inhibitory effect on *S. mutans* biofilm. In the case of a passive surface (polystyrene disk), it caused inhibition of biomass increase between 12 and 74 h (relative to sucrose by 0.016 AU (absorbance unit) and 0.022 vs. 0.006 AU for erythritol). Sorbitol had a similar inhibitory effect, with an average biomass increase of 0.016 AU. The opposite effect was observed in the case of xylitol, whose addition to the medium caused a sharp increase in the total biomass to the level of 0.050 AU (Table 2, Figure 2).

Table 2, Table 3 and Table 4 collect total biofilm masses as well as the amount of viable microorganisms in biofilms at all time-points, for all surfaces and sweeteners.

The total mass of biofilm formed by *S. mutans* was significantly lower (*p* < 0.0001) on both composite (Table 3) and glass ionomer materials (Table 4), at each time point, in the presence of sucrose and polyols compared to the control (polystyrene disk without dental material). Nevertheless, glass ionomer material had stronger inhibitory properties, which translated into a lower average biomass increase relative to control (mean for a substrate with sucrose 0.022 vs. 0.004). The addition of both sorbitol and erythritol had an additive effect, reducing the mass of a formed biofilm relative to sucrose. In the case of erythritol on both tested materials and for sorbitol on glass ionomer, a decrease in biomass below the initial value was observed between 12 and 72 h (pre-formed 12-h biofilm). In the case of xylitol, there was a decrease in biofilm formation on dental materials compared to the control surface. In the presence of xylitol, biofilm mass was higher than that of sucrose at all time-points.

### 3.5. Determination of the Amount of Live Microorganisms in a Biofilm

As with total biomass, the effect of erythritol caused the strongest effect of inhibiting the growth of live microorganisms (Figure 3). The growth of live microorganisms between 12 and 72 h for erythritol was equal to 0.03 log CFU/mL, while for sucrose this increase was equal to 0.32 log CFU/mL. Sorbitol was characterized by a weaker inhibitory effect (0.21 log CFU/mL increase) (Figure 3). Increased growth was observed for the medium enriched with xylitol (0.75 log CFU/mL).

A significant decrease in the live quantity of *S. mutans* in the biofilm formed on the surface of composite material in the case of a sucrose-enriched substrate was observed only between 12 and 48 h. Nevertheless, the addition of all three polyols caused a statistically significant reduction in the amount of CFU/mL. Erythritol was the most potent and completely inhibited the growth of microorganisms below baseline (pre-formed 12-h biofilm). Both xylitol and erythritol showed a life-reducing effect on both materials. On the other hand, xylitol on the glass ionomer surface showed an effect promoting the division of microorganisms more strongly than sucrose.

**Figure 4:**
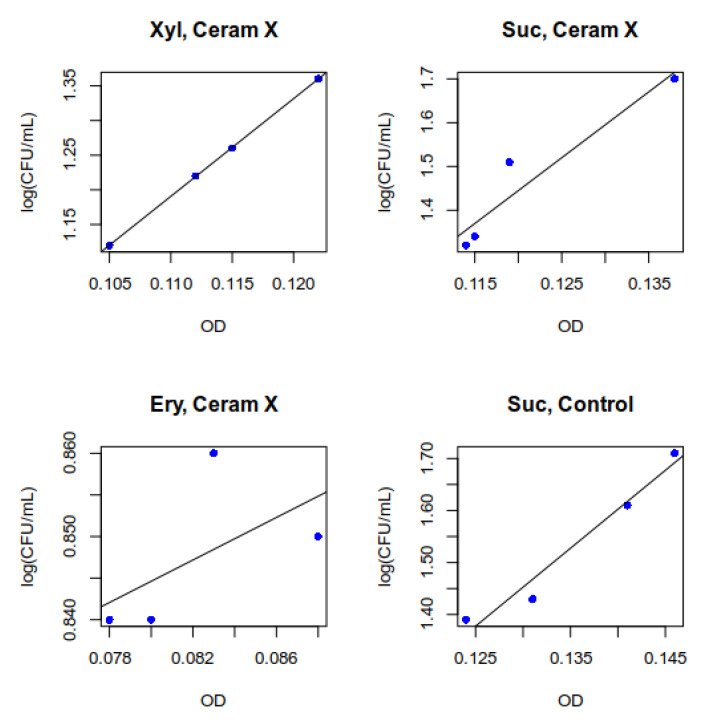
Examples of linear regression curves for four selected biofilms developed under influence of Xyl (xylitol), Suc (sucrose), and Ery (erythritol). High degree of dependence indicates that biomass increase is proportional to the increase in the number of viable *S. mutans* cells in the biofilm.

For most biofilms, a very strong relationship between total biomass and live microorganisms was found between 12 and 72 h. The strongest relationship occurred in the case of biofilms formed under the influence of xylitol on the composite material (r = 0.999) (Figure 4). A strong relationship was observed in the case of sucrose on the composite material (r = 0.792). A medium-strong relationship was identified in the case of erythritol on a composite substrate (r = 0.580). In other cases, the observed relationship was very strong (r = 0.9–1).

### 3.6. Morphological Characteristics of Biofilms

**Figure 5:**
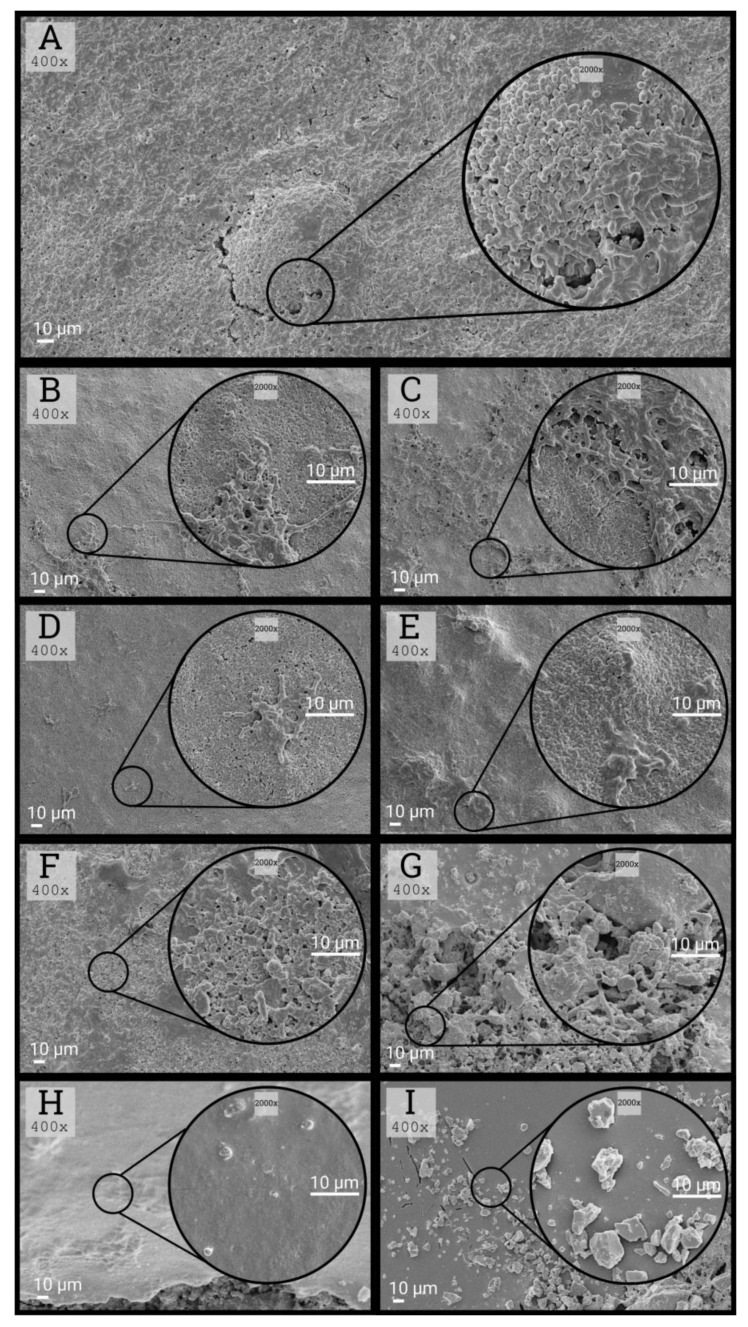
Micromorphology of obtained biofilms under scanning electron microscope after 24 h. Section (**A**): *S. mutans* on control surface in medium with 5% sucrose. Sections (**B**–**E**): biofilms formed on composite material in the presence of sucrose (**B**), xylitol (**C**), sorbitol (**D**), and erythritol (**E**). Sections (**F**–**I**): biofilms on glass ionomer material in the presence of sucrose (**F**), xylitol (**G**), sorbitol (**H**), and erythritol (**I**).

A stronger inhibitory effect on *S. mutans* was observed in the case of glass ionomers compared to composites, as shown in the images from the scanning electron microscope (Figure 5B–I). Erythritol was characterized by the best inhibitory properties among the tested sweeteners, which confirms the results obtained in bacterial biofilm culture. An example of the micromorphology of biofilms formed on a composite material under the influence of the studied polyols is shown in Figure 5B–E), while biofilms formed on a glass ionomer with the use of sweeteners are shown in Figure 5F–I.

## 4. Discussion

Dental caries is, among others, a result of dysbiosis of the oral microbiome with an elevated level of a number of cariogenic species (acidogenic and aciduric), including *S. mutans* (strong correlation), *Lactobacillus*, *Scardovia wiggsiae* from the *Bifidobacteriaceae* family, and several *Actinomyces* species, with *S. mutans* and *Streptococcus sobrinus* being the two most common species in humans [38,39,40,41,42,43,44].

Therefore, the present study used a single-species biofilm model based on clinical *S. mutans* isolates. The selection of clinical isolates was dictated by a different expression of the virulence factors of these strains related to the ability to form biofilm vs. reference strains [45]. *S. mutans* is one of over 700 species of oral microorganisms and is one of the main pathogens responsible for the development of caries [46], which is associated with its intensive metabolism of monosaccharides and disaccharides.

Dental treatment of carious lesions does not affect the composition of the oral microflora; therefore, in the absence of additional measures, further progression of caries in high-risk populations follows. One of the important aspects of controlling pathogenic biofilms seems to be the restoration of the oral microbiome balance, rather than elimination of a single pathological factor in the aspect of effective caries control and prevention [39].

One of the options for controlling pathological biofilms is the use of polyols. The current approach to the use of polyols in caries prevention focuses on their topical application in the form of chewing gums; lozenges; or in addition to toothpastes, dental floss and flakes or tissues for cleaning the mouth of the infant [47].

In the present study, the effect of 4 sweeteners used as food additives on biofilm formation on dental materials commonly used to fill defects in milk and permanent teeth was assessed. In the literature, xylitol, sorbitol, and erythritol have been described as non-cariogenic sweeteners, which have properties that inhibit the formation of cariogenic biofilms [12,48,49]. Sucrose, in turn, is considered to be the most cariogenic of sugars used in food production.

A number of in vitro studies indicate antimicrobial effect of xylitol [50,51,52,53]. These results are harder to observe in in vivo studies line described by Soderling et al. where xylitol consumption did not show reduction in the amount of *S. mutans* in participants’ saliva [54]. Reports show that short-term (≤6 months) daily intake of xylitol above 6 g reduces levels of *S. mutans*, while long-term (>12 months) use of xylitol has shown conflicting results [55].

There are numerous clinical studies assessing the effectiveness of xylitol, sorbitol, or erythritol supplementation in the prevention of caries in children, with results varying from no effect to significant caries reduction.

A meta-analysis of Riley et al. showed low-quality evidence suggesting that xylitol-containing fluoride toothpaste may be more effective than fluoride-only toothpaste in preventing tooth decay in children’s permanent teeth [47]. Other evidence is of low or very low quality and is insufficient to determine whether any other xylitol-containing products can prevent caries in children or adults [47].

Analysis of five randomized studies showed little effect of xylitol on the reduction of caries in children. Studies with higher doses of xylitol (> 4 g/day) showed an average reduction in caries, all of which are characterized by significant heterogeneity and very low quality of evidence [56].

Contrary to most published results, the present study did not show any cariostatic effect of xylitol; what is more, its stimulating effect exceeded that of sucrose on the production of biofilm biomass, and division of microorganisms was observed. This can be partly explained by the properties of wild strains that could potentially acquire xylitol resistance, which has been described by other researchers [57,58]. Nevertheless, attention is drawn to the fact that biofilms produced under the influence of this polyol were characterized by high biomass, whereas, as demonstrated by Lee et al., xylitol-resistant strains create biofilms with reduced density due to a decrease in GTF expression [58]. This issue requires further resolution in future research.

Another meta-analysis indicates moderate evidence of xylitol as the best-tested polyol for anti-caries effects when used topically at a dose of >4 g/day compared to other polyols or fluoride varnishes [48]. Studies have shown a better effect of erythritol in this area, which has a similar sweetness to sucrose, but it causes less severe laxogenic side effects [12]. These results are consistent with ours, where erythritol was proved to be the most effective in inhibiting the formation of *S. mutans* biofilm.

In a systematic review by Mickenautsch et al., clinical evidence demonstrating the effectiveness of xylitol compared to sorbitol in the context of anti-caries activity was proved to be contradictory and burdened with a high risk of error [48]. In the present study, sorbitol, similarly to erythritol, showed cariostatic properties; however, the strength of the effect was lower than in the case of the latter. Similar observations were made by other research teams, where the inhibitory in vitro effect on organic acid formation by *S. mutans* was dependent on oxygen partial pressure and eliminated in anaerobic conditions, which is associated with the process of incorporating sorbitol into glycolysis [59].

In this study, erythritol showed the strongest cariostatic properties. According to the review of in vivo studies, this polyol has the strongest anti-caries potential [12]. Makinen et al. showed a stronger inhibitory effect of erythritol on *S. mutans* growth (direct OD measurement in medium) than xylitol. A similar relationship was found in a clinical study, where volunteers used chewing gum and rinses based on xylitol and erythritol for six months (greater reduction in plaque index, salivary and plaque *S. mutans*, and fresh plaque weight for erythritol) [60].

The observed anti-biofilm effect exerted by sole dental materials (both composite and glass ionomer) is associated with the incorporation of antimicrobial substances into them. There are three categories of antimicrobial substances used for this purpose [61]. The first one includes enriching the material with water-soluble small molecules, such as chlorhexidine or benzalkonium chloride, diffusing within the material and neighboring tissues and providing protection against biofilm formation [62]. The second category includes monomers composed of two units, one prone to polymerization with the filling material and the other one with antimicrobial activity. The action of this group is limited only to the contact effect, without providing protection for neighboring tissues. An example of a monomer with antimicrobial activity is 12-methacryloyloxydodecypyridinium bromide (MDPB) [63]. The third group includes filler particles including nanoparticles of metals or their oxides [64]. Their action is based on the slow release of trace amounts of metals with antimicrobial properties, such as copper, silver, titanium, and zinc. This group also includes nano-ionomer fillers used in glass ionomer materials. Thanks to the use of ion-exchange glass releasing fluoride ions [64], the use of these materials in the prevention of secondary caries ensures effective antimicrobial activity and reduces susceptibility to demineralization of hard tooth tissues. Moreover, these particles are capable of binding fluoride ions delivered to the oral cavity as a part of fluoride prophylaxis [65].

Attention should be paid to significantly lower values of total biomass and the amount of live microorganisms in biofilms formed on surfaces of dental materials compared to the control surface. Surface roughness is one of the key factors determining the ability of primary colonizers to adhesion and biofilm formation [66]. Despite a greater roughness of glass ionomer, a stronger biofilm reduction was observed in its case, which may be related to its ability to prolong the release of fluoride ions [67]. These properties, as demonstrated in vitro in an artificial oral model, protect not only the glass ionomer surface and the surface tangent to it but also the tooth surface within at least 500 µm from the edge of the filling [68].

The role of *S. mutans* in the development of secondary caries is not limited to the degradation of healthy dental tissues. There are studies indicating the involvement of *S. mutans* and other oral microorganisms in the degradation of both composite and glass ionomer materials. The degradation of composite materials by these bacteria has been well described and is associated with the production of esterases responsible for hydrolysis of composite components [69,70,71]. The physiological role of esterases for *S. mutans* has not been thoroughly studied; however, it is associated with the virulence of this species [69]. In addition, degradation is also caused by cyclic drops in the mouth pH associated with food intake, in particular foods rich in sucrose [71]. Both of these processes are intensified in the biofilm environment, where there is an accumulation of organic acids and bacterial enzymes [70]. As a result, the described processes lead to changes in the composite microstructure, pore formation, which in turn is associated with weakening of the material and the appearance of micro-leaks and cracks exposing the dentin. At this stage, the formation of secondary defects at the material-tissue interface is accelerated and associated with difficult access for mechanical plaque removal and greater dentine susceptibility of enamel to demineralization (the process starts at pH < 6.7, while enamel demineralization efficiently occurs only when the pH drops below 5.5) [72,73].

The process of degradation of glass ionomer materials is characterized by a different mechanism is associated with the leaching of metal ions from the matrix and glass particles under the influence of acids, which then leads to the absorption of liquids and gradual dissolution [74]. Consequently, it comes to an increase in surface and surface roughness, which in turn leads to facilitated further development of the biofilm [75]. Evidence of the destructive effects of acids can be found in the study carried out by De Paula et al., who demonstrated an increase in roughness and a decrease in hardness of the glass ionomer material under the influence of acids from food in a sterile environment [74]. Size of the filler particles is the factor influencing chemical and mechanical resistance of glass ionomers. With a decrease of the particle diameter and with an increase of the packing level, the material resistance increases [74]. The final effect of biofilm microorganisms on glass ionomers is the loss of filling particles, loss of hardness, increase in roughness, and the appearance of micro-cracks [76,77,78].

Due to the documented impact of cariogenic biofilms on composite and glass ionomer materials, the replacement of sugars for non-cariogenic sweeteners seems to be important not only from the point of view of developing new demineralization outbreaks but also to protect physicochemical properties of these materials and more effectively prevent secondary caries.

There are limitations in this study that could be addressed in future research. Firstly, the study was based on using simplified single-species biofilm model based on clinical *S. mutans* isolates. Although over 700 different microbial species have been found in the mouth, *S. mutans* is one of the main pathogens responsible for the development of caries due to acid resistance and intensive metabolism of monosaccharides and disaccharides [46]. Although the in vitro model of a single-species biofilm is a great simplification of the actual prevailing conditions in vivo, it allows to ensure reproducible culture conditions, thus facilitating the analysis and interpretation of data. Future research should be extended to multi-species models that more closely reflect in vivo conditions in which interspecies interactions play a major role at both the genetic, metabolic, and structural levels. Secondly, in the present study, the microorganisms were exposed to sweeteners throughout the experiment, which does not reflect in vivo conditions, where salivary flow causes leaching of diet components, and the sweeteners themselves are given periodically. This problem could be solved by applying a biofilm model based on artificial mouth using alternate cycles of delivery and elution of the test substances.

In summary, polyols (including erythritol) can complement the basic methods of caries prevention due to the possibility of their use as sugar substitutes, oral microbiome modifiers, agents preventing the transfer of cariogenic bacteria, and compounds with both local (oral) and systemic benefits (prevention and treatment of obesity, diabetes, hypertension, and cardiovascular diseases). To date, only xylitol out of polyols was included in the recommendations of the AAPD (American Academy of Pediatric Dentistry) for children at moderate to high risk of caries, as well as by ADA (American Dental Association) for children from over 5 years of age, despite inconsistency of evidence indicating a significant reduction in *S. mutans* and caries in children [79]. On the other hand, EAPD (European Academy of Pediatric Dentistry) recommends the use of chewing gums containing xylitol by mothers during the eruption of milk teeth in their children in order to prevent early childhood caries [80].

## 5. Conclusions

Two (sorbitol and erythritol) of the three sucrose polyol substitutes tested showed a biofilm-inhibiting effect on the surface of the tested dental materials. Due to the documented impact of cariogenic biofilms on composite and glass ionomer materials, the replacement of sugars for non-cariogenic sweeteners seems to be important not only from the point of view of developing new demineralization outbreaks but also to protect physicochemical properties of these materials and more effectively prevent secondary caries.

Based on the obtained results and a review of the literature, the authors believe that the abandonment of sucrose as a food additive in favor of sorbitol and erythritol can be an effective way to complement the prevention of primary and secondary caries. Bearing in mind the results of the analysis of biofilms created on particular materials used to fill cavities in the group of patients of developmental age, the choice of a glass ionomer material in secondary caries prophylaxis has an additional protective anti-biofilm effect due to its ability to release fluoride ions.

In conclusion, our study showed for the first time that the use of erythritol and glass ionomers simultaneously in secondary caries prophylaxis increases their anti-caries effectiveness in the early phase of *S. mutans* mono-specific biofilm formation. The use of glass ionomers for dental fillings in children in combination with the replacement of ingested sugar with erythritol can be a promising element in the strategy of secondary caries prevention, but these conclusions should be confirmed by further studies on mixed biofilms and clinical trials.

## Figures and Tables

**Table 1:** Surface roughness analysis of dental materials after UV exposure in the presence of BHI culture medium with 5% sucrose, 5% xylitol, 5% sorbitol, or 5% erythritol. The results of Ra in µm are expressed as mean (+/−SD); different letters in superscript indicate significant (∝ = 0.05) differences between groups (Scheffe’s test).

Material	5% Sucrose	5% Xylitol	5% Sorbitol	5% Erythritol
Control	0.172 (0.030) ^a^	0.154 (0.012) ^a^	0.155 (0.010) ^a^	0.149 (0.011) ^a^
Composite	0.321 (0.031) ^a^	0.271 (0.033) ^a^	0.256 (0.023) ^a^	0.247 (0.014) ^b^
Glass ionomer	1.321 (0.024) ^a^	1.121 (0.025) ^a^	0.967 (0.018) ^a^	0.887 (0.053) ^b^

Note: The same letter in superscript in line means that there is no significant differences between marked groups. Different letters mean that there is significant difference between groups (*p* < 0.05).

**Table 2:** Total biofilm biomass and number of viable microorganisms in biofilms formed on control surface (polystyrene disc) in media with different sweetening agents.

Character	Sucrose	Xylitol	Sorbitol	Erythritol	(ANOVA) *p*
**Time**	**OD540 mean ± SD (median ± QD)**				
12 h	0.11 ± 0.01 (0.11 ± 0.01)	0.10 ± 0.01 ** (0.11 ± 0.01)	0.10 ± 0.01 **** (0.10 ± 0.01)	0.09 ± 0.01 **** (0.09 ± 0.01)	<0.0001
24 h	0.12 ± 0.01 (0.12 ± 0.00)	0.11 ± 0.02 (0.12 ± 0.01)	0.10 ± 0.01 *** (0.11 ± 0.01)	0.08 ± 0.01 **** (0.08 ± 0.01)	<0.0001
48 h	0.12 ± 0.01 (0.12 ± 0.01)	0.11 ± 0.02 (0.12 ± 0.01)	0.11 ± 0.01 ** (0.11 ± 0.01)	0.08 ± 0.01 **** (0.08 ± 0.00)	<0.0001
72 h	0.14 ± 0.01 (0.14 ± 0.01)	0.12 ± 0.01 **** (0.13 ± 0.01)	0.11 ± 0.01 **** (0.12 ± 0.01)	0.08 ± 0.01 **** (0.08 ± 0.01)	<0.0001
	**Log(CFU/mL) mean ± SD (median ± QD)**				
12 h	1.32 ± 0.05 (1.32 ± 0.05)	1.12 ± 0.07 *** (1.13 ± 0.06)	0.92 ± 0.10 *** (0.93 ± 0.05)	0.85 ± 0.12 *** (0.85 ± 0.08)	<0.0001
24 h	1.34 ± 0.04 (1.34 ± 0.02)	1.22 ± 0.05 *** (1.22 ± 0.03)	1.14 ± 0.09 *** (1.15 ± 0.08)	0.86 ± 0.22 *** (0.90 ± 0.12)	<0.0001
48 h	1.51 ± 0.16 (1.48 ± 0.09)	1.26 ± 0.06 *** (1.25 ± 0.03)	1.24 ± 0.07 *** (1.24 ± 0.04)	0.84 ± 0.21 *** (0.85 ± 0.08)	<0.0001
72 h	1.70 ± 0.07 (1.71 ± 0.03)	1.36 ± 0.06 *** (1.35 ± 0.04)	1.31 ± 0.07 *** (1.30 ± 0.04)	0.84 ± 0.10 *** (0.85 ± 0.06)	<0.0001

One-way ANOVA with post hoc Tukey test, ** *p* < 0.01; *** *p* < 0.001; **** *p* < 0.0001, SD–standard deviation.

**Table 3:** Total biofilm biomass and number of viable microorganisms in biofilms formed on CeramX composite material in media with different sweetening agents.

Character	Sucrose	Xylitol	Sorbitol	Erythritol	(ANOVA) *p*
**Time**	**OD540 mean ± SD (median ± QD)**				
12 h	0.08 ± 0.01 (0.08 ± 0.01)	0.07 ± 0.01 (0.07 ± 0.01)	0.06 ± 0.01 **** (0.06 ± 0.01)	0.06 ± 0.01 **** (0.06 ± 0.00)	<0.0001
24 h	0.08 ± 0.00 (0.08 ± 0.00)	0.07 ± 0.01 ** (0.07 ± 0.01)	0.06 ± 0.01 **** (0.06 ± 0.01)	0.06 ± 0.01 **** (0.06 ± 0.00)	<0.0001
48 h	0.08 ± 0.01 (0.08 ± 0.00)	0.07 ± 0.01 (0.07 ± 0.01)	0.06 ± 0.01 **** (0.06 ± 0.01)	0.06 ± 0.01 **** (0.05 ± 0.01)	<0.0001
72 h	0.08 ± 0.00 (0.08 ± 0.00)	0.08 ± 0.01 (0.08 ± 0.01)	0.06 ± 0.01 **** (0.06 ± 0.01)	0.05 ± 0.01 **** (0.05 ± 0.01)	<0.0001
	**Log(CFU/mL) mean ± SD (median ± QD)**				
12 h	1.26 ± 0.09 (1.26 ± 0.05)	1.31 ± 0.09 (1.31 ± 0.05)	0.97 ± 0.16 **** (0.95 ± 0.13)	0.83 ± 0.13 **** (0.84 ± 0.10)	<0.0001
24 h	1.27 ± 0.06 (1.26 ± 0.05)	1.33 ± 0.08 (1.34 ± 0.05)	1.10 ± 0.15 **** (1.15 ± 0.15)	0.80 ± 0.11 **** (0.81 ± 0.09)	<0.0001
48 h	1.28 ± 0.05 (1.29 ± 0.03)	1.32 ± 0.07 (1.32 ± 0.05)	1.17 ± 0.12 **** (1.20 ± 0.10)	0.77 ± 0.12 **** (0.75 ± 0.09)	<0.0001
72 h	1.28 ± 0.05 (1.29 ± 0.03)	1.35 ± 0.06 ** (1.36 ± 0.05)	1.15 ± 0.11 **** (1.18 ± 0.08)	0.74 ± 0.12 **** (0.73 ± 0.09)	<0.0001

One-way ANOVA with post hoc Tukey test, ** *p* < 0.01; **** *p* < 0.0001, SD–standard deviation.

**Table 4:** Total biofilm biomass and number of viable microorganisms in biofilms formed on Equia Forte Fil glass ionomer material in media with different sweetening agents.

Characteristic	Sucrose	Xylitol	Sorbitol	Erythritol	(ANOVA) *p*
**Time**	**OD540 mean ± SD (median ± QD)**				
12 h	0.12 ± 0.00 (0.12 ± 0.00)	0.13 ± 0.01 (0.13 ± 0.00)	0.12 ± 0.00 (0.12 ± 0.00)	0.10 ± 0.01 **** (0.11 ± 0.01)	<0.0001
24 h	0.13 ± 0.01 (0.13 ± 0.00)	0.14 ± 0.01 **** (0.14 ± 0.01)	0.13 ± 0.01 (0.13 ± 0.00)	0.11 ± 0.01 **** (0.11 ± 0.00)	<0.0001
48 h	0.14 ± 0.00 (0.14 ± 0.00)	0.15 ± 0.01 **** (0.15 ± 0.01)	0.13 ± 0.01 ** (0.13 ± 0.00)	0.11 ± 0.01 **** (0.11 ± 0.01)	<0.0001
72 h	0.15 ± 0.01 (0.15 ± 0.00)	0.18 ± 0.02 **** (0.17 ± 0.01)	0.14 ± 0.01 ** (0.14 ± 0.00)	0.11 ± 0.01 **** (0.11 ± 0.01)	<0.0001
	**Log(CFU/mL) mean ± SD (median ± QD)**				
12 h	1.39 ± 0.14 (1.39 ± 0.07)	1.49 ± 0.15 ** (1.47 ± 0.06)	1.31 ± 0.12 (1.31 ± 0.08)	1.33 ± 0.12 (1.33 ± 0.07)	<0.0001
24 h	1.43 ± 0.09 (1.43 ± 0.05)	1.64 ± 0.19 **** (1.57 ± 0.07)	1.36 ± 0.11 (1.35 ± 0.06)	1.34 ± 0.12 * (1.35 ± 0.07)	<0.0001
48 h	1.61 ± 0.13 (1.64 ± 0.11)	1.85 ± 0.19 **** (1.85 ± 0.11)	1.46 ± 0.15 **** (1.44 ± 0.08)	1.35 ± 0.12 **** (1.36 ± 0.07)	<0.0001
72 h	1.71 ± 0.12 (1.73 ± 0.07)	2.24 ± 0.29 **** (2.23 ± 0.16)	1.53 ± 0.15 *** (1.51 ± 0.05)	1.36 ± 0.13 **** (1.38 ± 0.08)	<0.0001

One-way ANOVA with post hoc Tukey test, * *p* < 0.05; ** *p* < 0.01; *** *p* < 0.001; **** *p* < 0.0001, SD–standard deviation.

## Appendix A

In the case of tested polyols, their potential inhibitory mechanism on dental plaque was presented. Sucrose, which is a cariogenic sugar, is digested into glucose and fructose, which are included in the glycolysis process associated with obtaining energy and substrates for further synthesis by bacteria. The product of glycolysis is pyruvate, which in subsequent transformations is metabolized to organic acids responsible for chemical digestion of tooth hard tissues. Sorbitol (Sor) can be included in the glycolysis via sorbitol-6-phosphate dehydrogenase (Sor-6-P-DH) resulting in the generation of excess (relative to glucose/fructose) NADH molecules. High NADH/NAD ratio in the cell can be equalized by increased lactate synthesis by lactic acid dehydrogenase and under hypoxic conditions by triggering the puryvate–formate lyase (PFL) pathway, which is associated with the production of acetic acid, formic acid, and ethanol. Under aerobic conditions, PFL is inhibited, resulting in a rapid increase in the NADH/NAD ratio, which leads to inhibition of glycolysis at a stage of 3-phosphoglycerylaldehyde dehydrogenase (GLAPDH), protecting the cell from excess NADH. Xylitol (Xyl) transported to the cytoplasm is phosphorylated at the expense of the phosphate group derived from phosphoenolpyruvate (PEP) transferred by the HPr and EI cytoplasmic enzymes. Xylitol-5-phosphate (Xyl-5-P) cannot be included in glycolysis and accumulates, leading to cell vacuolization. It can also be actively removed outside the cell, which is associated with energy loss. A detailed model of erythritol interaction with *S. mutans* has not been described so far; however, its activity is associated with inhibition of expression of enzyme genes’ encoding, e.g., extracellular proteases depriving microorganisms of access to free amino acids and glucose/fructose transferases participating in the synthesis of the biofilm matrix.
